# Clinical Usefulness of Right Ventricle–Pulmonary Artery Coupling in Cardiovascular Disease

**DOI:** 10.3390/jcm12072526

**Published:** 2023-03-27

**Authors:** Qing He, Yixia Lin, Ye Zhu, Lang Gao, Mengmeng Ji, Li Zhang, Mingxing Xie, Yuman Li

**Affiliations:** 1 Department of Ultrasound Medicine, Union Hospital, Tongji Medical College, Huazhong University of Science and Technology, Wuhan 430022, China; 2 Hubei Province Clinical Research Center for Medical Imaging, Wuhan 430022, China; 3 Hubei Province Key Laboratory of Molecular Imaging, Wuhan 430022, China

**Keywords:** right ventricular–pulmonary artery coupling, pulmonary arterial hypertension, heart failure, valvular heart disease, hypertension

## Abstract

Right ventricular–pulmonary artery coupling (RV-PA coupling) refers to the relationship between RV contractility and RV afterload. Normal RV-PA coupling is maintained only when RV function and pulmonary vascular resistance are appropriately matched. RV-PA uncoupling occurs when RV contractility cannot increase to match RV afterload, resulting in RV dysfunction and right heart failure. RV-PA coupling plays an important role in the pathophysiology and progression of cardiovascular diseases. Therefore, early and accurate evaluation of RV-PA coupling is of great significance for a patient’s condition assessment, clinical decision making, risk stratification, and prognosis judgment. RV-PA coupling can be assessed by using invasive or noninvasive approaches. The aim of this review was to summarize the pathological mechanism and evaluation methods of RV-PA coupling, the advantages and disadvantages of each method, and the application value of RV-PA coupling in various cardiovascular diseases.

## 1. Introduction

In recent years, there has been increasing appreciation for the importance of right ventricular (RV) coupling to the pulmonary arterial (PA) circulation, which is called right ventricular–pulmonary artery coupling (RV-PA coupling). RV-PA coupling refers to the relationship between RV contractility and RV afterload and is defined as the ratio of RV end-systolic elastance (contractility index) to PA elastance (arterial load index) [1,2,3,4,5], which is considered to be the gold standard assessment of RV-PA coupling [6,7,8]. RV function depends on the complex interplay between myocardial contractility and pulmonary vascular afterload [6,9,10]. Normal RV-PA coupling is maintained only when cardiac function and vascular resistance are appropriately matched [11]. At the setting of increased RV afterload, there is preserved RV-PA coupling if RV contractility has increased through RV hypertrophy and adaptation to the load to the level where RV function (cardiac index, tricuspid annular plane systolic excursion (TAPSE), right ventricular ejection fraction (RVEF), etc.) is maintained within a normal range. RV-PA uncoupling occurs when RV contractility cannot increase to match RV afterload, resulting in RV dysfunction and right heart failure [1]. In chronic RV pressure overload, RV-PA uncoupling is considered as the driving cause of RV maladaptation and ultimate RV failure [12]. Therefore, it has important clinical implications for the timely detection of high-risk patients, decision making, and improving the long-term survival of patients. The aim of this review was to summarize pathological mechanism and evaluation methods of RV-PA coupling, the advantages and disadvantages of each method, and the application value of RV-PA coupling in various cardiovascular diseases.

## 2. Measurement of Right Ventricular–Pulmonary Artery Coupling

RV-PA coupling is a comprehensive index that requires an overall understanding of RV function and RV afterload, and can be assessed by using integrated hemodynamic parameters related to RV function and afterload [2]. RV-PA coupling is influenced by RV function; however, it is very challenging to achieve a good assessment of RV function [13]. RV-PA coupling can be measured by using invasive and noninvasive ways. The invasive measurement is right heart catheterization (RHC), and noninvasive methods include echocardiography and cardiac magnetic resonance (CMR) imaging. Several measurements for RV-PA coupling are described below. The advantages and disadvantages of each measurement are summarized in Table 1.

### 2.1. End-Systolic Elastance/Effective Arterial Elastance

Previous studies have shown that multi-beat end-systolic elastance (Ees)/effective arterial elastance (Ea) from invasive pressure–volume loops by RHC has been regarded as the gold standard for assessment of RV-PA coupling [1,14,15,16,17,18], as other alternative indicators assume a linearity and zero-crossing of end-systolic pressure–volume relationships. Ees is equivalent to cardiac contractility and Ea represents ventricular afterload [19]. Ees is defined as end-systolic pressure (ESP) divided by ventricular end-systolic volume (ESV), which is considered as a load-independent parameter of myocardial contractility [19,20]. Ea is defined as the ratio of ESP divided by the stroke volume (SV) and incorporates all factors of total ventricular afterload, involving peripheral resistance, arterial compliance, and characteristic impedance, and it is a mechanical characteristic that reflects the afterload system functionally [21]. The optimal Ees/Ea ratio is between 1.5 and 2.0, as this can maximize efficiency and minimize energy expenditure [22,23]. This ratio is still preserved even in the early stages of diseases because of the adaptation to the pressure overload results in concentric RV hypertrophy, which is characterized by an increased wall thickness with almost no change in the chamber volume; thus, RV Ees matches the elevated Ea. Only with progressive RV decompensation does RV Ees begin to fall with an inability to match an increase in Ea; Ees/Ea declines, the mechanical work efficiency will be low, and lastly, RV-PA coupling becomes uncoupled [20]. The methods for measuring Ees/Ea include single-beat and multiple-beat. At present, multiple-beat methods are more valuable in practice. Uncertainties in maximum pressure (Pmax) calculations and the imperfect linearity of end-systolic pressure–volume relationships may result in disagreement between single-beat and multiple-beat methods [24]. The advantages of Ees/Ea in assessing RV-PA coupling are its high sensitivity and accuracy [6,25]. However, it also has various defects, which include that it is invasive and technically demanding, expensive, and unpractical at the bedside [15]. Moreover, both Ees and Ea may become abnormal with disease progression, but the Ees/Ea ratio may be close to normal instead. To surmount the above limitations, more and more researchers have explored other accessible, convenient, noninvasive alternatives to measuring RV-PA coupling.

### 2.2. Noninvasive Methods

#### 2.2.1. Tricuspid Annular Plane Systolic Excursion/Pulmonary Arterial Systolic Pressure

**Figure 1:**
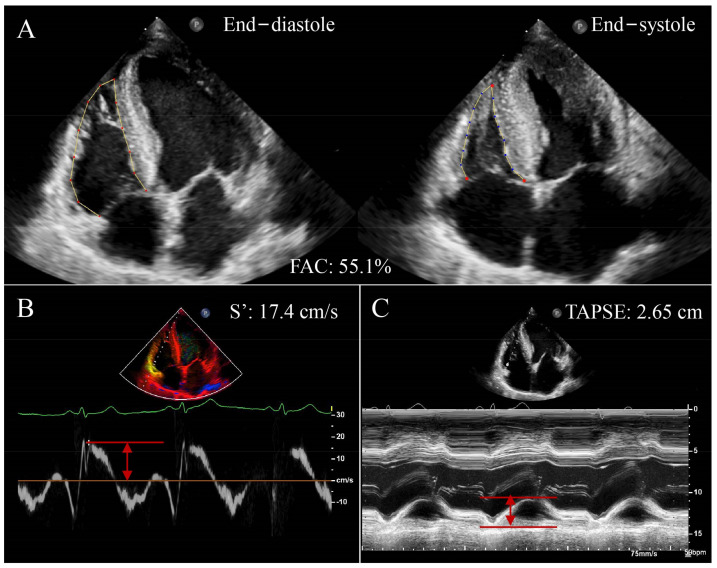
Conventional RV function parameters using two-dimensional echocardiography in a patient with HFrEF. (**A**) RVFAC; (**B**) S’; (**C**) TAPSE; RV, right ventricular; HFrEF, heart failure with reduced ejection fraction; RVFAC, right ventricular fractional area change; S’, tricuspid annular systolic velocity;TAPSE, tricuspid annular plane systolic excursion. Red arrows represent measurements for parameters.

TAPSE is the most frequently used echocardiographic parameter of RV systolic function in clinical settings (Figure 1A). Pulmonary arterial systolic pressure (PASP), as a Doppler index of RV afterload, is also easily acquired by echocardiography. TAPSE/PASP, as a method for the noninvasive measurement of RV-PA coupling, has gradually become an alternative parameter for Ees/Ea [26,27,28,29,30,31,32]. It was shown that TAPSE/PASP was strongly correlated with the invasive assessment of RV-PA coupling [15]. A decreased TAPSE/PASP ratio suggests the decoupling of RV contractility from its afterload [21,33,34,35]; the cut-off value for abnormally low TAPSE/PASP was 0.36 mm/mmHg, below which RV-PA coupling was considered compromised. TAPSE is easy to obtain, being independent of image quality and highly reproducible [36]. However, TAPSE is limited by angle dependence and less accuracy in the case of severe tricuspid regurgitation (TR) [37]. In addition, TAPSE represents only the longitudinal movement of the basal segment of the RV free wall and may fail to accurately reflect the entire RV function, especially in patients with RV regional wall motion abnormalities. Increasing numbers of studies have applied TAPSE/PASP to assess the balance between RV function and pulmonary circulation, and it has become a simple, noninvasive, and effective surrogate of Ees/Ea.

#### 2.2.2. Tricuspid Annular Systolic Velocity/Right Ventricular Systolic Pressure

Tricuspid annular systolic velocity (S’) measured by tissue Doppler imaging is another highly reproducible indicator of longitudinal RV contractility of the basal RV free wall (Figure 1B). S’/right ventricular systolic pressure (RVSP) integrates both RV systolic function and RV afterload, and therefore, it can be used to evaluate RV-PA coupling. A reduced S’/RVSP has been reported as a vital prognostic marker associated with high mortality in cardiac intensive care patients and could be used for risk stratification of critically ill patients; in addition, S’/RVSP highly correlates with TAPSE/PASP [38]. S’ is easily obtained and not affected by image quality, and it depends less on the afterload than TAPSE. Yet, it is angle-dependent. In addition, it assumes that the function of a single RV segment represents the entire RV function, which is less accurate in RV infarction or pulmonary embolism. RVFAC, S’, and TAPSE using two-dimensional echocardiography are shown in Figure 1.

#### 2.2.3. Right Ventricular Fractional Area Change/Right Ventricular Systolic Pressure

Right ventricular fractional area change (RVFAC) reflects RV longitudinal and transverse systolic function and is calculated as (RV end-diastolic area—RV end-systolic area)/RV end-diastolic area × 100% (Figure 1C) [39]. The ratio of RVFAC/RVSP can be used as a noninvasive method to assess RV-PA coupling. The RVFAC/RVSP ratio has been confirmed to be of superior prognostic value compared with RV systolic function (TAPSE or RVFAC) alone [40]. RVFAC is highly correlated with RVEF and has been reported as a simple and rapid method to evaluate RV systolic function. The RVFAC/RVSP ratio is easy to acquire, angle-independent, and does not require additional analysis software [41]. Nevertheless, RVFAC measurement requires the delineation of RV endocardial borders, and RVFAC accuracy may be compromised when image quality is poor.

#### 2.2.4. Stroke Volume/End-Systolic Volume

As mentioned earlier, Ees is calculated as ESP/ESV and Ea is calculated as ESP/SV. Both have a common ESP, so the RV-PA coupling ratio can be further simplified as SV/ESV, which is an alternative volume-based method to RV-PA coupling assessment [42,43]. In a study cohort of pediatric pulmonary arterial hypertension (PAH), which indicated the feasibility of the SV/ESV ratio derived from CMR, it was found that it correlated with the RV-PA coupling ratio evaluated by cardiac catheterization [44]. SV/ESV has been demonstrated to have application value in dilated cardiomyopathy (DCM), PAH, and other diseases [42,44,45,46]. The SV/ESV ratio can also be derived from three-dimensional echocardiography (3 DE). The 3 DE determined SV/ESV ratio has been shown to have a strong correlation with right-heart-catheterization-derived RV-PA coupling in adults with PAH [46]. The limitation of the SV/ESV ratio is that it is a simplified Ees/Ea and may underestimate Ees/Ea in practice [47,48]. Compared with Ees/Ea, SV/ESV assumes the linearity and zero-crossing of end-systolic pressure–volume relationships, which may result in moderately increased bias [24].

#### 2.2.5. Right Ventricular Longitudinal Strain/Right Ventricular Systolic Pressure

**Figure 2:**
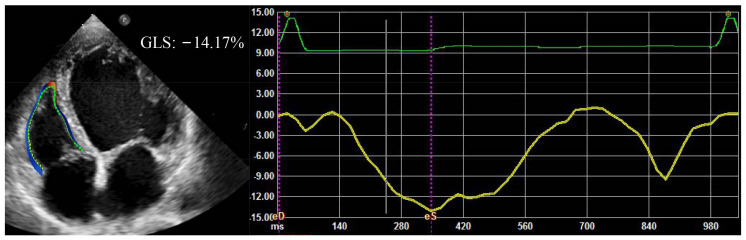
RVGLS in a patient with HFrEF. RVGLS, right ventricular global longitudinal strain.

Recently, right ventricular longitudinal strain (RVLS) obtained by two-dimensional speckle tracking echocardiography has been demonstrated to be a more sensitive indicator for RV function than conventional echocardiographic parameters, and it can detect subclinical myocardial dysfunction earlier [49]. The assessment of right ventricular global longitudinal strain is displayed in Figure 2. Therefore, the ratio of RVLS to PASP corresponds to the ratio of RV systolic function to RV afterload, and has been proposed to correlate with the Ees/Ea ratio [50,51,52]. RVLS is noninvasive and angle-independent, has excellent accuracy and reproducibility, and is not affected by surrounding tissue structure and respiratory movement. However, two-dimensional speckle tracking echocardiography is hampered by image quality, 2D plane measurement, and the out-of-plane motion of speckles [53].

#### 2.2.6. Right Ventricular Ejection Fraction/Pulmonary Arterial Systolic Pressure

**Figure 3:**
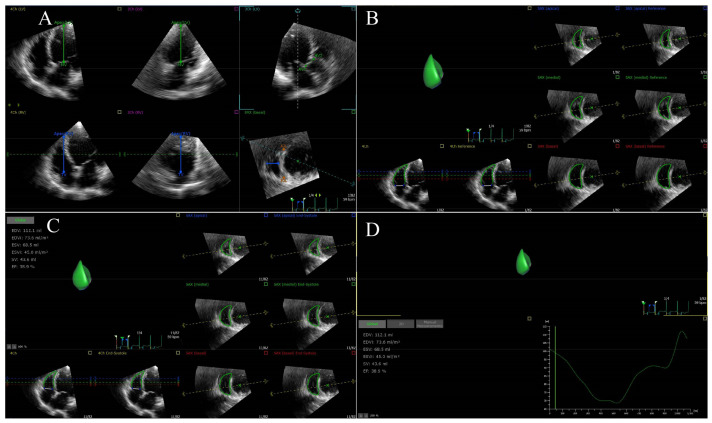
RVEF using 3 DE in a patient with HFrEF. (**A**) Setting reference points; (**B**) RV endocardial border identification and tracking at end-diastole; (**C**) RV endocardial border identification and tracking at end-systole; (**D**) RVEF is automatically generated. RVEF, right ventricular ejection fraction; 3 DE, three-dimensional echocardiography.

RVEF is the most common used index of RV systolic function in everyday clinical practice and is strongly predictive of adverse outcomes in patients with a variety of cardiovascular diseases [54,55]. It has been confirmed that a lower RVEF/PASP ratio was associated with an increased risk of heart failure (HF) or death [37]. RVEF can be measured by 3 DE and CMR, and it does not rely on the RV geometry assumption. The analysis of RVEF using 3 DE is shown in Figure 3. However, 3 DE is hindered by a lower temporal resolution and is dependent on the image quality [56].

## 3. Clinical Applications of RV-PA Coupling

### 3.1. Pulmonary Arterial Hypertension

The level of clinical treatment continues to improve, but the quality of life and prognosis of PAH patients are still unsatisfying [56,57]. PAH leads to RV pressure overload due to increased PVR and is unable to maintain normal RV cardiac output; thereby, it results in the decoupling of RV-PA and ultimate RV failure and death [58,59,60,61]. RV-PA coupling is crucial for the risk stratification of PAH patients [2,62,63]. In a study involving 26 patients with PAH, they underwent CMR, RHC, and RV pressure–volume assessment with multi-beat (MB) determination of Ees/Ea on the same day, and follow-up to observe their disease development [6]. The investigators found 16 subjects eventually met the criteria for clinical worsening, and they were more likely to have a lower MB Ees/Ea ratio (0.7 ± 0.5 versus 1.3 ± 0.8, *p* = 0.02), and MB Ees/Ea predicted the time to clinical worsening in PAH. In addition, MB Ees/Ea was shown to be superior to RVEF and SV/ESV in its ability to predict CW in human PAH [6]. RV-PA coupling was relatively maintained in earlier stages but was impaired markedly with more severe PAH in 139 adults with PAH [42]. In the above study, contractility was augmented to match the increased load and maintained the SV in the early phases of chronic PAH, preserving optimal coupling at the expense of suboptimal mechanical efficiency. Ultimately, RV failed as a pump, which indicated inadequate coupling and reduced myocardial efficiency. This study demonstrated that the evaluation of RV-PA coupling may help to expound the mechanisms of RV failure and to identify patients at risk, or guide the timing of therapeutic interventions in PAH [42]. In a study of 203 patients with PAH, RV volume, RVEF, RVLS, and the RVEF/PASP ratio were measured using 3D echocardiography [37]. Their findings showed that RV-PA coupling declined with the advancing World Health Organization functional class (WHO-FC). Patients with WHO-FC I and II had a significantly higher RVEF/PASP ratio than patients with WHO-FC III and IV (both *p* < 0.001). Additionally, PAH is one of the most important factors for the evaluation of patients before heart transplant, which is associated with prognosis after heart transplant [64]. In a study cohort of 44 heart transplant recipients, it was shown that RV-PA coupling is impaired early after heart transplant and improves significantly from 7 to 30 days post-transplant, and the evolution is correlated with donor–recipient size matching. The interaction between RV-PA coupling and donor–recipient size matching may play an important role in preventing major RV dysfunction in patients post-heart transplant [65]. Thus, the evaluation of RV-PA coupling is beneficial for the early identification of high-risk patients with PAH and the clinical management of patients as well as the prediction of outcomes.

### 3.2. Heart Failure

HF is a clinical syndrome in which symptoms and/or signs are caused by structural and/or functional abnormalities of the heart, as evidenced by elevated levels of brain natriuretic peptide and/or objective evidence of pulmonary or systemic congestion [66,67,68,69,70]. HF can be divided into three types: heart failure with preserved ejection fraction (HFpEF), heart failure with mid-range ejection fraction (HfmrEF), and heart failure with reduced ejection fraction (HfrEF) [66,70,71,72]. Considerable evidence has demonstrated that assessment of RV-PA coupling plays a crucial role in risk stratification, monitoring efficacy, and predicting outcomes in HF patients [14,31,73]. Since there are few studies on HfmrEF at present, the application of RV-PA coupling in HF mainly focuses on the following two aspects.

#### 3.2.1. Heart Failure with Preserved Ejection Fraction

HfpEF accounts for about half of HF patients [74], and it was previously thought to be mainly a disorder of left ventricular (LV) diastolic function [75,76,77,78,79,80,81,82]. Previous researches have revealed that both pulmonary circulation and RV function are impaired in HfpEF patients [83,84,85,86,87,88]. In a study cohort of 67 patients with HfpEF, which applied Ees/Ea to evaluate RV-PA coupling in HfpEF patients at rest and during exercise, the results showed that RV-PA coupling was significantly lower in HfpEF patients at rest compared with the controls [89]. Ees/Ea was elevated in HfpEF patients during the initial stage of exercise, which may be associated with a significant increase in RV contractility, but RV-PA coupling worsened during peak exercise. Evidence shows that RV-PA coupling is a favorable indicator of exercise tolerance in HfpEF patients and can assist in guiding treatment to improve exercise tolerance [89]. A study including 384 patients with HfpEF indicated that TAPSE was associated closely with PASP in HfpEF patients, and combining the information on RV function and pulmonary artery pressures in a single TAPSE/PASP ratio allowed us to obtain an accurate risk stratification in all HF patients [33]. Further research that involved 387 patients with HfpEF compared RV contractile function and RV-PA coupling in HfpEF patients with variable disease severity stratified according to TAPSE/PASP ratio tertile (1: <0.35; 2: 0.35 to 0.57; 3: >0.57) [26]. They found that patients in tertile 1 presented with higher PASP and RV end-diastolic and end-systolic areas, and lower TAPSE and RVFAC. Their results demonstrated that the TAPSE/PASP ratio was inversely correlated with the New York Heart Association functional class and could independently predict adverse outcomes. Therefore, the TAPSE/PASP ratio seems helpful in unmasking the early occurrence of symptoms and an initial depression in the RV functional reserve [26]. In a prospective research study, HfpEF patients had a decreased RV systolic reserve and abnormal RV-PA coupling in the early stage of disease, but the aforementioned parameters were largely reversible [90]. RV-PA coupling was enhanced with β-adrenergic stimulation in HfpEF subjects, which confirmed that interventions targeting RV function or afterload may be beneficial for patients at an early stage of disease progression [90]. Additionally, investigators studied the occurrence of pulmonary interstitial edema in patients with HfpEF, and assessed RV-PA coupling using three indicators: TAPSE/PASP, S’/PASP, and RVFAC/RVSP [91]. The above study revealed that three indicators were reduced in patients with HfpEF compared with the healthy controls. These findings demonstrated that pulmonary interstitial edema may be associated with abnormal RV-PA coupling, and early measurement of RV-PA coupling could predict the occurrence of pulmonary interstitial edema in HfpEF patients [91]. In conclusion, the evaluation of RV-PA coupling may be crucial to identify patients at higher risk, delay disease progression, and improve the prognosis for patients with HfpEF.

#### 3.2.2. Heart Failure with Mid-Range Ejection Fraction

The pathological process of HfrEF is similar to HfpEF, but it has a high incidence as well as poor prognosis. HfrEF is often accompanied by PAH, which can significantly affect disease progression and prognosis; thus, it is crucial to evaluate RV-PA coupling in patients with HfrEF. In a study cohort of 112 patients with HfrEF, Ees/Ea was closely associated with RV function and predicted the overall survival of HfrEF patients [92]. Furthermore, the best cut-off for Ees/Ea to discriminate overall survival was 0.68, and patients with Ees/Ea < 0.68 were characterized by significantly lower intrinsic RV contractility and higher afterload parameters (Ea or PVR) than those with Ees/Ea ≥ 0.68. Hence, Ees/Ea evaluation was beneficial for a better clinical risk stratification and decision-making process for the timing of LV assist devices and/or heart transplantation in patients with HfrEF [92]. In a study including 205 HfrEF patients who underwent an overall assessment with echocardiography and exercise tolerance tests, a lower TAPSE/PASP was correlated with lower functional class, exercise capacity, and ventilatory inefficiency, and was predictive of adverse outcomes among patients with HfrEF [93]. Hence, the TAPSE/PASP ratio would be a helpful screening marker to identify patients with HfrEF with severely impaired exercise tolerance and triage them to a specific cardiopulmonary exercise test as needed [93]. In a cohort of 54 patients with HfrEF undergoing cardiac resynchronization therapy, it was demonstrated that TAPSE/PASP displayed good sensitivity (90%) and specificity (81.8%) for predicting the response to cardiac resynchronization therapy. Moreover, a lower TAPSE/PASP ratio was associated with a higher incidence of adverse cardiovascular events during the follow-up [94]. Hence, these findings suggested that the TAPSE/PASP ratio may become a standard echocardiographic evaluation parameter for HfrEF patients undergoing cardiac resynchronization therapy.

### 3.3. Hypertension

Compared to the left heart, a few studies have investigated RV function in hypertensive patients. Actually, long-term overload of the left ventricle can result in increased pressure and injury, with intimal fibrosis and remodeling on the pulmonary vessels, leading to elevated PVR, PA pressure, and RV afterload, and ultimately right ventricular dysfunction came up [95]. Patients with uncontrolled and untreated hypertension have worse RV and atrial mechanics, as well as functional capacity, in comparison with the better treated hypertensive patients [96]. In a study of 446 hypertensive patients with different glucose tolerance levels, patients were graded as the normal glucose tolerance 1 group with 1 h post-load plasma glucose < 155 mg/dL, the NGT 2 group with 1 h post-load plasma glucose ≥ 155 mg/dL, the impaired glucose tolerance group, and the type 2 diabetes mellitus group, from low to high according to 1 h post-load plasma glucose. In this study, the value of PASP and PVR were significantly higher from the first to the fourth group, as well as a reduction in RV function parameters demonstrated by lower TAPSE/PASP, TAPSE, and RVFAC. Not only the left heart but also the RV was affected by early glucose metabolism disorders. Monitoring RV-PA coupling in hypertensive patients could provide timely identification of right heart injury caused by impaired glucose tolerance, and improve long-term survival [97]. Furthermore, RV-PA coupling assessed by TAPSE/PASP was compared between the control group and the hypertensive group [98]. They found that TAPSE/PASP in the hypertensive group was significantly lower than that in control group [98]. Thus, accurate assessment of RV-PA coupling could be helpful to reduce the mortality, assist patients with treatment, and decrease the incidence of cardiovascular-related events.

### 3.4. Valvular Heart Disease

The tricuspid valve is closely related to RV and PA. A study investigating 1149 patients with secondary TR applied TAPSE/PASP to estimate whether RV-PA coupling could improve risk stratification and showed that 470 patients had a decreased TAPSE/PASP ratio; that is, decoupling of RV-PA, and patients with decoupled RV-PA had a higher incidence of symptoms and comorbidities, with more severe TR and RV remodeling [99]. In addition, in a series of 444 TR patients undergoing transcatheter tricuspid valve repair or replacement (TTVR), multivariable Cox regression analysis showed that a higher TAPSE/PASP ratio was associated with a declined risk of all-cause mortality, and the magnitude of TR reduction after TTVR was independently related with a reduction in post-TTVR RV-PA coupling. These results revealed that RV-PA coupling may be helpful to patient selection and prognostication following TTVR [100]. A study that involved 372 patients with HF and severe secondary mitral regurgitation reported that patients with a lower RVLS/RVSP ratio had larger mean proximal isovelocity surface area-derived effective regurgitant orifice areas and were more likely to have severe (4+) mitral regurgitation [50]. By multivariable analysis, impaired RV-PA coupling was strongly associated with enhanced risk for death or hospitalization in patients with HF and secondary mitral regurgitation. Patients with RV-PA uncoupling experienced more New York Heart Association functional class III or IV symptoms compared with those with normal RV-PA coupling [50]. A study evaluated S *‘*/RVSP by echocardiography to investigate RV-PA coupling in patients with aortic stenosis who experienced transcatheter aortic valve replacement, and they found that S’ was increased in aortic stenosis patients after transcatheter aortic valve replacement, indicating enhanced RV-PA coupling [101]. This study demonstrated that S’/RVSP could monitor the changes in condition of patients with aortic stenosis, clarify the efficacy of transcatheter aortic valve replacement, and facilitate the timely adjustment of treatment [101]. Thus, RV-PA coupling plays a key role in risk stratification, the prediction of clinical outcomes, and in guiding treatment in patients with valvular heart disease.

### 3.5. Congenital Heart Disease

Tetralogy of Fallot (TOF) is the most frequent cyanotic congenital heart disease. Patients with repaired TOF commonly develop RV dilatation, which can cause progressive RV failure. Previous studies primarily focused on the evaluation of RV dimensions and function, but RV is coupled to the low-pressure PA system with high compliance [21]. Therefore, the RV and pulmonary artery should be assessed as a whole in patients with repaired TOF. In a retrospective study of 129 adults (repaired TOF (*n* = 84) and VPS with previous intervention (*n* = 45)), RV-PA coupling was lower in the TOF group FAC/RVSP (ratio 1.10 ± 0.29 vs. 1.48 ± 0.22 (*p* < 0.001)) and TAPSE/RVSP ratio (0.51 ± 0.15 vs. 0.78 ± 0.11 (*p* < 0.001)) compared to the healthy controls because of a higher RV afterload (RVSP 42 ± 3 mm Hg vs. 31 ± 3 mmHg (*p* = 0.012)) [40]. Similar results were seen in the VPS group. A reduction in RV-PA coupling caused by a disproportionate increase in PA elastance highlighted the important role of abnormal PA elastic properties and its potential interaction with RV-PA coupling in TOF and VPS patients with PR [40]. Additionally, investigators aimed to determine the association between noninvasive RV-PA coupling indices (TAPSE/RVSP and FAC/RVSP ratio) and disease severity in TOF patients, and compared this association to peak oxygen consumption [102]. They found that RV-PA coupling was modestly correlated with peak oxygen consumption in patients with TOF. Their findings highlighted the importance of noninvasive RV-PA coupling indices as a potential supplementary tool in the evaluation of patients with TOF [102]. By evaluating RV-PA coupling, a study including 24 patients provided new insights in searching for additional mechanisms for the deterioration in RV performance and the response to therapeutic interventions in the long-term follow-up of patients with repaired TOF [21]. This study elucidated the emerging role of RV-PA coupling as a contributing mechanism for the decline in RV function and impaired effect to inotropic drugs in patients with TOF [21]. In a retrospective study of 135 patients with post-operative TOF, the data showed that RV-PA uncoupling was prevalent in repaired TOF patients, and the uncoupling group had obvious lower TAPSE/PASP compared with the coupling group [19]. Furthermore, their results indicated that the TAPSE/PASP ratio came out as the strongest predictor for RV-PA uncoupling, helping to provide additional understanding of the decline in RV performance [19]. Thus, RV-PA coupling exerts a major influence on risk stratification and outcome prediction in patients after TOF.

### 3.6. Cardiomyopathy

DCM is characterized by the presence of systolic dysfunction and LV or biventricular dilatation and in the absence of coronary artery disease or abnormal loading conditions, which frequently has a genetic background [103]. Recently, the long-term survival of DCM patients has increased significantly. It remains the most common reason for heart transplant in the Western world nevertheless [104]. Originally, the RV adapts by remodeling and hypertrophy, leading to an initial increase in contractility. RV maladaptation occurs with disease progression, and the RV begins to dilate, followed by the decoupling of RV-PA [65]. Another study investigated the RV-PA coupling defined as the SV/ESV ratio in 139 outpatients with DCM, and reported that RV-PA coupling was significantly more impaired with increasing symptom severity, and was the only independent determinant of severe DCM, regardless of age, diuretic use, LV systolic function, LV diastolic function, and PASP [45]. Therefore, RV-PA coupling was further proposed as a marker of disease severity in DCM patients. One of the earliest studies to assess RV-PA coupling as an outcome predictor in DCM, which included 60 patients with DCM who were followed for a mean period of 18 months, revealed that noninvasive RV-PA coupling was more significantly impaired in patients with DCM who were rehospitalized for HF exacerbation, and the RVLS/PASP ratio and the RVEF/PASP ratio were independent predictors of rehospitalizations. The cut-off values for RVLS/PASP and RVEF/PASP were –0.40 and −1.30, respectively [105]. Hirasawa et al. demonstrated that a low TAPSE/SPAP ratio was associated with adverse outcomes in hypertrophic cardiomyopathy (HCM), and RV-PA coupling could be important in risk stratification of patients with HCM [106].

## 4. Summary and Prospect

Decoupling of RV-PA is the terminal state of various cardiovascular diseases and poses a great harm to the health of patients. Therefore, early and accurate evaluation of RV-PA coupling in patients has important clinical value for the early identification of high-risk patients, delaying disease progression, guiding treatment, and improving prognosis. At present, various methods for RV-PA coupling assessment have been employed, including invasive and noninvasive approaches. The gold standard measurement of RV-PA coupling is Ees/Ea obtained by RHC, which is invasive, complex, and not widely available. It is expected to replace invasive techniques as the preferred method in the future with the continuous development of easy and noninvasive techniques such as Large sample, and prospective studies are needed to clarify the optimal application parameters of the noninvasive estimation of RV-PA coupling in order to help doctors to provide better management of patients.

## Figures and Tables

**Table 1:** Advantages and disadvantages of methods evaluating RV-PA coupling.

Index		Advantage	Disadvantage
**Invasive**	Ees/Ea (gold standard)	High sensitivity and accuracy.	High technical difficulty;
			Limited bedside application;
			False normality;
			Expensive.
**Noninvasive**	TAPSE/PASP	Easily obtained;	Angle dependence;
		Not reliant on image quality;	Low accuracy in case of TR;
		Reproducible.	Inability to reflect overall RV function.
	RVLS/RVSP	Angle independence;	Relies on image quality;
		High sensitivity and accuracy;	Presence of out-of-plane motion of speckles.
		Reproducible;	
		Unaffected by surroundings.	
	RVFAC/RVSP	Easily obtained;	Depends on image quality.
		Angle independence;	
		Does not need software analysis.	
	RVEF/PASP	Does not rely on the RV geometry assumption.	Low temporal resolution on 3 DE;
			Depends on image quality.
	SV/ESV	High reliability	May underestimate real RV-PA coupling.
	S’/RVSP	Easily obtained;	Angle dependence.
		Independent of image quality;	Uses single segment to represent overall RV function.
		Less dependence on afterload than TAPSE.	

Ees, end-systolic elastance; Ea, arterial elastance; TAPSE, tricuspid annular plane systolic excursion; PASP, pulmonary arterial systolic pressure; TR, tricuspid regurgitation; RV, right ventricle; RVLS, right ventricular longitudinal strain; RVSP, right ventricular systolic pressure; RVFAC, right ventricular fractional area change; RVEF, right ventricular ejection fraction; 3 DE, three-dimensional echocardiography; SV, stroke volume; ESV, end-systolic volume; RV-PA coupling, right ventricular–pulmonary artery coupling; S’, tricuspid annular systolic velocity.

## Data Availability

Not applicable.
